# Nonlinear relationship between sleep duration and non-suicidal self-injurious behaviour among Chinese adolescents

**DOI:** 10.1186/s12888-021-03539-x

**Published:** 2021-10-21

**Authors:** Ying Tang, Yuhui Wan, Shaojun Xu, Shichen Zhang, Jiahu Hao, Fangbiao Tao

**Affiliations:** 1 Department of Maternal, Child and Adolescent Health, School of Public Health, Anhui Medical University, No 81 Meishan Road, Hefei, 230032 Anhui China; 2 MOE Key Laboratory of Population Health Across Life Cycle, No 81 Meishan Road, Hefei, 230032 Anhui China; 3 NHC Key Laboratory of Study on Abnormal Gametes and Reproductive Tract, No 81 Meishan Road, Hefei, 230032 Anhui China; 4 Anhui Provincial Key Laboratory of Population Health and Aristogenics, Anhui Medical University; No 81 Meishan Road, Hefei, 230032 Anhui China

**Keywords:** Sleep duration, Non-suicidal self-injury, Adolescents, Students

## Abstract

**Background:**

Previous studies have shown a positive association between sleep deprivation and non-suicidal self-injury (NSSI) among adolescents, but few studies have described the effects of oversleeping and weekend catch-up sleep on NSSI. The present study aimed to explore the nonlinear relationship between sleep duration and NSSI among Chinese adolescents.

**Methods:**

Data from China’s National Adolescent Health Surveillance for the years 2014 to 2015 were collected from 15,713 students located across four provinces in China. A self-report questionnaire was used to assess sleep duration and 12-month NSSI. Binomial logistic regression models were used to examine the association between NSSI and sleep duration. The locally estimated scatter plot smoothing (LOESS) method was used to explore the associations of total NSSI number with sleep duration, and binomial regression analysis was used to test this relationship.

**Results:**

About 68.5% of adolescents reported sleeping less than 8 h on weeknights, while 37.8% of adolescents slept more than 10 h per night during weekends. The 12-month prevalence rate of NSSI was 29.4%. Compared to adolescents who reported weekend catch-up sleep of 0–1 h, those who slept < 0 h (adjusted odds ratio [*aOR*] = 1.38, 95% Confidence Interval [95% *CI*]: 1.16–1.64) had a higher risk of NSSI. Males who reported ≥3 h of weekend catch-up sleep had significantly increased odds of NSSI (*aOR* = 1.20, 95%*CI*: 1.01–1.42). Notably, a positive U-shaped association was observed between the sleep duration and the total NSSI number.

**Conclusions:**

The findings reveal a nonlinear relationship between sleep duration and NSSI among Chinese adolescents. Therefore, it is necessary to be vigilant and screen for sleep duration among adolescents in NSSI treatment or prevention.

**Supplementary Information:**

The online version contains supplementary material available at 10.1186/s12888-021-03539-x.

## Background

Non-suicidal self-injury (NSSI) is defined as the direct, deliberate destruction of one’s own body tissue without suicidal intent [1]. NSSI is a significant global public health issue among adolescents with high prevalence rates [2]. Previous results demonstrate that the rates of NSSI in youth significantly vary between countries as well as across different cities and districts within a country [3–5]. Worldwide, the average 12-month prevalence of NSSI is 19.0% [6]. In China, the average 6–24 months prevalence of NSSI was 23.3% [7]. NSSI leads to both physical and social-emotional harm in the short and long term, and may be a gateway for suicide, as NSSI is associated with both an increased desire and capability for suicide [8]. Given the consequences of NSSI and suicide on both society and individuals, accurate identification of the risk factors associated with NSSI is critical.

Adequate sleep is necessary for both physical and mental health among adolescents. However, sleep deprivation is prevalent in modern society [9]. The National Sleep Foundation observed that approximately 61% of American adolescents aged 13–18 years reported sleeping less than the recommended 8–10 h of sleep [10–12]. In a survey of 585 adolescents from a high school in China, the vast majority of students (over 93%) slept less than 8 h during weeknights, with over 42% sleeping less than 6.5 h [13]. In another study, approximately 51.0% of 4801 Chinese adolescents (aged 11–20 years) received less than 8 h of sleep during weeknights [14]. Adolescence is a period characterized by dramatic changes in cognitive, behavioral, social, and emotional functioning [15]. The onset of adolescence has also been characterized by changes in sleep/wake patterns. Both sleep/wake pattern changes and changes to the sleep-pressure system (sleep homeostasis) during adolescence favor later timing of sleep [16]. These changes, combined with prevailing social pressures, are responsible for most teenagers sleeping too late and too little [16].

Studies have shown that while school schedules require adolescents to be fully awake early in the morning, reduced parental control over bedtime, sleep hygiene such as caffeine consumption, high levels of social media use, and other behavioral patterns often shape adolescent lifestyles toward predominantly nighttime behavior [17–19]. This interaction leads to chronic patterns of sleep deprivation, a tendency for higher rates of daytime sleepiness, and accumulation of sleep debt during the school week [20]. Adolescents typically attempt to oversleep on non-school days to compensate their sleep debt, especially by sleeping on weekend mornings [20]. In a survey of 9567 secondary school students in New Zealand, the bedtime and rise times during weekdays were 22:17 and 06:57; on the weekend, their bedtime and rise times were 00:09 and 09:31 [21]. In another study, Lee et al. found that the mean sleep duration of 3785 middle and high school students in Korea was 7 h on weekdays, 8.9 h on weekends, and 1.8 h for weekend catch-up sleep [22]. A survey of 1629 Chinese adolescents (aged 12 to 19 years) showed that the average bedtime on school nights was 23:24 and total sleep time was 7.3 h. During weekends, the average bedtime and rise time were delayed by 64 min and 195 min, respectively [23]. This phenomenon of short, early sleep on weekdays and later, longer sleep on weekends has been called weekend sleep lag or social jet lag [24, 25].

Inadequate sleep duration, sleepiness, and irregular sleep patterns may lead to poor academic performance, psychological symptoms such as depression, and physical health problems such as headaches, obesity and cardiometabolic dysfunction [26]. The effect of sleep patterns on risk-taking behaviors has also been a cause of concern in recent years. Several reports have suggested that sleep duration is associated with the NSSI in adolescents. In a study of Swedish adolescents, poor sleep was associated with NSSI among girls, but not among boys [27]. In a study of Norwegian adolescents, insomnia, short sleep duration, long sleep onset latency, wake after sleep onset, and large differences between weekdays versus weekends bedtimes yielded higher odds of self-harm consistent with a dose–response relationship [28]. Previous studies have focused on sleep quality and inadequate sleep duration on NSSI; however, few studies have focused on oversleeping and weekend catch-up sleep [29, 30]. To date, there has been no evidence of a U-shaped relationship between sleep duration and NSSI.

A previous study showed that adolescents with psychological symptoms are at an elevated risk of developing NSSI [31]. In addition, insufficient sleep in adolescents can be affected by excessive screen time use, with insufficient sleep in adolescents having been linked to suicidal ideation/NSSI [32–34]. Therefore, in this study, we aimed to explore the nonlinear relationship between sleep duration and NSSI among Chinese adolescents, considering the influence of sleep quality, psychological symptoms and screen time. We hope that this study will help us provide relevant strategies to support adolescent NSSI prevention.

## Methods

### Participants

The data were obtained from China’s National Adolescent Health Surveillance from 2014 to 2015. This is an annual school-based surveillance system involving adolescents and young adults from the same junior and senior high schools located in Xinxiang (central), Yangjiang (south), Chongqing (west) and Shenyang (north) areas. These areas are broadly representative of the average population within China in terms of economic development and demographic composition. Eight schools (2 junior and 2 senior high schools from urban and rural, respectively) in each geographic region were randomly selected.

A total sample of 15,713 students from grade 7–12 were invited to participate in the study. Of these students, 1037 (6.6%) were excluded from the study because of (1) absence from school on the day of the survey or unwillingness to respond to the questionnaire, (2) missing data through fictitious or inconsistent responses. Finally, we received 14,676 (93.4%) effective questionnaires, including 7017 males (47.8%) and 7377 junior school students (50.3%). The students aged from 10 to 24 years (mean 15.20, SD 1.75). In addition, the participants of four regions were 3968 (Xinxiang), 3539 (Yangjiang), 4007 (Chongqing), and 3162 (Shenyang), respectively.

### Procedure

The study ensured that all the participants and their guardians were aware of the purpose and content of this investigation. The students who agreed to participate in the survey stayed in the classroom, while those who did not were permitted to leave. Each center used an anonymous, self-reported questionnaire. The completion of the questionnaire took approximately 25 min. A teacher was present in the classroom, but was unable to observe student responses. The investigators checked the accuracy and completeness of the returned questionnaires and removed the unqualified questionnaires. Informed consent was obtained from the parents/guardians of each student prior to the completion of questionnaires. Approval for the design and data collection procedures was obtained by the Ethics Committee of Anhui Medical University. Detailed survey information can be found in our previous article [35].

### Measures

#### Socio-demographic information

A self-report questionnaire was used to collect sociodemographic information, including sex (male or female), grade (junior or senior level in middle school), urban/rural residency, household structure (only child or more than one child), parents’ education level (completion of junior middle school, senior middle school, college, etc.) and self-perceived family economic status (poor, general or good). The additional file provided a questionnaire (Additional File 1).

#### Psychological symptoms and screen time

Psychological symptoms, including emotional symptoms (depression and anxiety symptoms, e.g., ‘Do you always feel distressed?’), behavioural symptoms (paranoid and hostile behaviors, e.g., ‘Do you always have the impulse to damage something?’) and social adaptation symptoms (bad relationships with family and friends, e.g., ‘Could you not fit well into school life?’), were evaluated using the psychological domain of the Multidimensional Sub-health Questionnaire of Adolescents (MSQA) [31]. The MSQA has been widely used in mainland China and has been reported by various groups to be a valid and reliable method to explore the current state of psychological health in adolescents [36, 37]. Cronbach’s alpha for the MSQA was 0.951 in the present study.

Screen time was measured by video watching and video game playing times. The subjects reported screen time using the following questions: “On an average school day, for how many hours do you watch videos (such as watching TV, mobile phone, MP4, DVD/VCD)?” and “On an average school day, for how many hours do you play video games (such as game consoles, computer games, mobile games)?”. A similar question was used to define the average screen time on weekends [38]. All of these questions have seven answer categories: ‘0 h’, ‘≤0.5 h’, ‘0.5–1 h’, ‘1–2 h’, ‘2–4 h’, ‘4–6 h’, and ‘>6 h’.

#### Sleep variables

The survey contained two questions concerning sleep duration [35]. The first question represented the usual weekday sleep duration: “In the last month, how many hours of actual sleep did you usually get at night on weekdays?” A similar question was used to define the usual weekend sleep duration. Daily sleep duration was calculated as a weighted average of weekday and weekend sleep durations using the following formula: ([{usual weekday sleep duration} × 5] + [{usual weekend sleep duration} × 2])/7. Weekend catch-up sleep was calculated using the formula: ([usual weekend sleep duration]–[usual weekday sleep duration]). For the purpose of statistical analyses in the present study, sleep duration was divided into 6 categories: ‘< 6 h’, ‘6–7 h’, ‘7–8 h’, ‘8–9 h’, ‘9–10 h’, and ‘≥10 h’. Weekend catch-up sleep was divided into 5 categories: ‘< 0 h’, ‘0–1 h’, ‘1–2 h’, ‘2–3 h’, and ‘≥3 h’. Sleep quality was ascertained from responses on a 4-point Likert scale to the question “In the past month, what did you think of your sleep quality?” Response options were very good, good, poor, and very poor [35].

#### NSSI

NSSI was assessed using the following question: ‘In the past 12 months, have you ever harmed yourself in a way that was deliberate, but not intended to take your life?’ Eight NSSI behaviors were presented, and the details of the questions were as follows: (1) Have you ever hit yourself?; (2) Have you ever pulled your hair yourself?; (3) Have you ever banged your head or fist against something?; (4) Have you ever pinched or scratched yourself?; (5) Have you ever bitten yourself?; (6) Have you ever cut or pierced yourself?; (7) Have you ever deliberately taken an overdose (e.g. of drugs, alcohol or smoking)?; and (8) Have you ever ingested a toxic substance or object? For those who confirmed that they had engaged in NSSI, the frequency of NSSI was investigated [31]. In this study, for statistical analysis purposes, NSSI was analyzed in two different ways. Frequency of self-harm was coded as follows: (a) 2 groups: ‘NSSI’ (one or more times), and Non-NSSI (none); and (b) continuous variable: ‘Total number of self-injuries’ (sum of the eight types of frequency of self-harm). In the present study, the Cronbach’s alpha coefficient for the NSSI was 0.798.

### Statistical analysis

Statistical analyses were carried out using SPSS version 24.0, and R software version 3.6.1. Means and standard deviations of sleep duration were calculated separately for each sex group as well as for each grade group of participants. For comparisons between sex and grade groups, independent *t*-tests were conducted for sleep duration, and effect sizes were calculated. The frequencies and percentages of NSSI in the different groups were calculated. Pearson’s chi-squared test was used to examine differences in sex, grade, sleep duration and other variables between adolescents who reported NSSI and those who did not, and effect sizes were calculated. Univariate logistic regression analysis was used to examine the associations between covariates (e.g., sex, sleep duration, etc.) with NSSI. Binomial logistic regression models were used to examine the associations of NSSI with daily sleep duration, weekday sleep duration, weekend sleep duration and weekend catch-up sleep, by adjusting for sociodemographic variables, etc. Sex and grade differences in these associations were examined. The locally estimated scatter plot smoothing (LOESS) method was used to explore the associations of total NSSI number with daily sleep duration, weekday sleep duration, weekend sleep duration and weekend catch-up sleep. This method was especially useful for representing the nonlinear relationship between variables, for example, the U-shaped relation. GetData Graph Digitizer version 2.26 was used to find the nadir point. Binomial regression analysis was used to test the relationship between total NSSI number and sleep duration by adjusting for sociodemographic variables, sleep quality, psychological symptoms, and screen time. The level of significance was set at *P* <  0.05.

## Results

### Comparisons of sleep duration between sex and grade groups

The usual daily sleep duration of adolescents (the total group) was 7.6 h and the mean sleep duration was 7 h on weekdays, 9 h on weekends, and 1.9 h for weekend catch-up sleep. Males had longer weekday sleep durations than females did, while females had longer weekend sleep durations and weekend catch-up sleep than males did. Junior middle school students slept significantly longer than senior middle school students on both weekdays and weekends, and had less weekend catch-up sleep than senior middle school students did (*P* <  0.001 for each, see Table 1). The percentage of adolescents who slept < 8 h per night during weeknights was 68.5%, and the percentage of adolescents who slept≥10 h per night during weekends was 37.8% for the total group (see Table 2). In this survey, about 63.5% of males and 73.1% of females reported sleeping for less than 8 h on weeknights. Approximately 49.6% of junior middle school students and 87.6% of senior middle school students reported sleeping for less than 8 h on weeknights.

**Table 1:** **Comparisons of sleep duration between sex and grade groups**

Variables	Daily sleep duration(h)	Weekday sleep duration(h)	Weekend sleep duration(h)	Weekend catch-up sleep(h)
Sex				
Male(*n* = 7017)	7.64 ± 1.12	7.15 ± 1.26	8.87 ± 1.72	1.72 ± 1.86
Female(*n* = 7659)	7.51 ± 1.01	6.91 ± 1.13	9.01 ± 1.57	2.10 ± 1.70
*t*	7.251	11.941	−5.209	−12.816
*P* value	< 0.001^**^	< 0.001^**^	< 0.001^**^	< 0.001^**^
*d*	0.129	0.212	−0.089	− 0.224
Grade				
Junior middle school (*n* = 7377)	7.95 ± 1.09	7.50 ± 1.21	9.08 ± 1.58	1.59 ± 1.66
Senior middle school(*n* = 7299)	7.20 ± 0.91	6.55 ± 0.99	8.81 ± 1.70	2.26 ± 1.86
*t*	45.460	51.745	10.169	−22.953
*P* value	< 0.001^**^	< 0.001^**^	< 0.001^**^	< 0.001^**^
*d*	0.824	0.960	0.159	−0.360
Total (*N* = 14,676)	7.58 ± 1.07	7.03 ± 1.20	8.95 ± 1.64	1.92 ± 1.79

* *P* <  0.05, ** *P* <  0.001; Statistical methods: independent *t*-tests, *d* is effect sizes

**Table 2:** Prevalence of NSSI by sample characteristics

Variables	N(%)	NSSI (%)	Non-NSSI (%)	*χ*^2^	*φ/V*
Regional areas				21.170^**^	0.038^**^
YangJiang	3539(24.1)	1103(31.2)	2436(68.8)		
ShenYang	3162(21.5)	935(29.6)	2227(70.4)		
XinXiang	3968(27.0)	1060(26.7)	2908(73.3)		
ChongQing	4007(27.3)	1219(30.4)	2788(69.6)		
Sex				35.341^**^	−0.049^**^
Male	7017(47.8)	2228(31.8)	4789(68.2)		
Female	7659(52.2)	2089(27.3)	5570(72.7)		
Grade				22.538^**^	−0.039^**^
Junior middle school	7377(50.3)	2301(31.2)	5076(68.8)		
Senior middle school	7299(49.7)	2016(27.6)	5283(72.4)		
Urban/rurality				0.001	0.000
Rural	7493(51.1)	2205(29.4)	5288(70.6)		
Urban	7183(48.9)	2112(29.4)	5071(70.6)		
Only child				6.727^*^	−0.021^*^
Yes	6608(45.0)	2015(30.5)	4593(69.5)		
No	8068(55.0)	2302(28.5)	5766(71.5)		
Father’s education level				11.609^*^	0.028^*^
Less than junior middle school	2158(14.7)	689(31.9)	1469(68.1)		
Junior middle school	6239(42.5)	1840(29.5)	4399(70.5)		
Senior middle school	4256(29.0)	1186(27.9)	3070(72.1)		
College or more	2023(13.8)	602(29.8)	1421(70.2)		
Mother’s education level				15.825^*^	0.033^*^
Less than junior middle school	2686(18.3)	867(32.3)	1819(67.7)		
Junior middle school	6331(43.1)	1854(29.3)	4477(70.7)		
Senior middle school	4027(27.4)	1119(27.8)	2908(72.2)		
College or more	1632(11.1)	477(29.2)	1155(70.8)		
Family economic status				62.899^**^	0.065^**^
Poor	2020(13.8)	741(36.7)	1279(63.3)		
General	10,860(74.0)	3036(28.0)	7824(72.0)		
Good	1796(12.2)	540(30.1)	1256(69.9)		
Sleep quality				254.451^**^	0.132^**^
Very Good	2416(16.5)	457(18.9)	1959(81.8)		
Good	6426(43.8)	1788(27.8)	4638(72.7)		
Poor	4500(30.7)	1544(34.3)	2956(65.6)		
Very poor	1334(9.1)	528(39.6)	806(60.6)		
Psychological symptoms				906.125^**^	0.248^**^
Yes	4027(27.4)	1926(47.8)	2101(52.2)		
No	10,649(72.6)	2391(22.5)	8258(77.5)		
Weekday video time (h)				74.449^**^	0.071^**^
0	6331(43.1)	1701(26.9)	4630(73.1)		
≤ 0.5	3090(21.1)	874(28.3)	2216(71.7)		
0.5–1	2198(15.0)	696(31.7)	1502(68.3)		
1–2	1387(9.5)	438(31.6)	949(68.4)		
2–4	761(5.2)	258(33.9)	503(66.1)		
4–6	376(2.6)	139(37.0)	237(63.0)		
>6	533(3.6)	211(39.6)	322(60.4)		
Weekend video time (h)				167.631^**^	0.107^**^
0	1737(11.8)	362(20.8)	1375(79.2)		
≤ 0.5	1818(12.4)	475(26.1)	1343(73.9)		
0.5–1	2276(15.5)	618(27.2)	1658(72.8)		
1–2	2601(17.7)	727(28.0)	1874(72.0)		
2–4	2484(16.9)	784(31.6)	1700(68.4)		
4–6	1692(11.5)	574(33.9)	1118(66.1)		
>6	2068(14.1)	777(37.6)	1291(62.4)		
Weekday video game time (h)				32.089^**^	0.047^**^
0	8334(56.8)	2347(28.2)	5987(71.8)		
≤ 0.5	2770(18.9)	817(29.5)	1953(70.5)		
0.5–1	1486(10.1)	457(30.8)	1029(69.2)		
1–2	891(6.1)	274(30.8)	617(69.2)		
2–4	472(3.2)	160(33.9)	312(66.1)		
4–6	285(1.9)	93(32.6)	192(67.4)		
>6	438(3.0)	169(38.6)	269(61.4)		
Weekend video game time (h)				123.035^**^	0.092^**^
0	2790(19.0)	698(25.0)	2092(75.0)		
≤ 0.5	2354(16.0)	635(27.0)	1719(73.0)		
0.5–1	2173(14.8)	560(25.8)	1613(74.2)		
1–2	2330(15.9)	671(28.8)	1659(71.2)		
2–4	1820(12.4)	613(33.7)	1207(66.3)		
4–6	1392(9.5)	475(34.1)	917(65.9)		
>6	1817(12.4)	665(36.6)	1152(63.4)		
Daily sleep duration (h)				55.717^**^	0.062^**^
< 6	712(4.9)	284(39.9)	428(60.1)		
6–7	3343(22.8)	1036(31.0)	2307(69.0)		
7–8	5771(39.3)	1688(29.2)	4083(70.8)		
8–9	3359(22.9)	911(27.1)	2448(72.9)		
9–10	1145(7.8)	309(27.0)	836(73.0)		
≥ 10	346(2.4)	89(25.7)	257(74.3)		
Weekday sleep duration (h)				43.605^**^	0.055^**^
< 6	1125(7.7)	413(36.7)	712(63.3)		
6–7	4386(29.9)	1305(29.8)	3081(70.2)		
7–8	4546(31.0)	1354(29.8)	3192(70.2)		
8–9	3182(21.7)	854(26.8)	2328(73.2)		
9–10	1020(7.0)	284(27.8)	736(72.2)		
≥ 10	417(2.8)	107(25.7)	310(74.3)		
Weekend sleep duration (h)				49.429^**^	0.058^**^
< 6	287(2.0)	130(45.3)	157(54.7)		
6–7	716(4.9)	244(34.1)	472(65.9)		
7–8	1376(9.4)	431(31.3)	945(68.7)		
8–9	3729(25.4)	1054(28.3)	2675(71.7)		
9–10	3020(20.6)	860(28.5)	2160(71.5)		
≥ 10	5548(37.8)	1598(28.8)	3950(71.2)		
Weekend catch-up sleep (h)				59.123^**^	0.063^**^
< 0	966(6.6)	365(37.8)	601(62.2)		
0–1	1998(13.6)	529(26.5)	1469(73.5)		
1–2	3101(21.1)	825(26.6)	2276(73.4)		
2–3	4032(27.5)	1174(29.1)	2858(70.9)		
≥ 3	4579(31.2)	1424(31.1)	3155(68.9)		
Total	14,676(100.0)	4317(29.4)	10,359(70.6)		

* *P* <  0.05, ** *P* <  0.001; Statistical methods: Chi-square test, *φ*/*V* is effect sizes. NSSI is non-suicidal self-injury

### The prevalence rates and characteristics of NSSI

The 12-month prevalence rate of NSSI was 29.4%. Of all the self-injuries disclosed, the type of NSSI most reported included banging head/ fisting against the subject (18.4%), hitting (13.8%) and pinching/scratching (11.1%). A significant difference was found in the distribution of NSSI by most sample characteristics, including regional areas, sex, grade, household structure, parents’ education level, self-perceived family economic status, sleep quality, psychological symptoms and screen time (*P* <  0.05), while NSSI revealed no statistically significant differences by urban/rural residency (*P* > 0.05). In addition, there was a marked difference between sleep duration and catch-up sleep with NSSI (*P* <  0.001, see Table 2).

### Univariate logistic regression analyses

Univariate logistic regression analyses showed that the regional areas (crude odds ratios [*ORs*] = 0.83, 95% Confidence Interval [95% *CI*]: 0.76–0.92), sex (*ORs* = 1.24, 95%*CI*: 1.16–1.33), grade (*ORs* = 1.19, 95%*CI*: 1.11–1.28), only child (*ORs* = 1.10, 95%*CI*: 1.02–1.18), mother’s education level (*ORs* = 1.15, 95%*CI*: 1.01–1.32), self-perceived family economic status (*ORs* = 1.35, 95%CI: 1.18–1.54), sleep quality (*ORs* = 0.36, 95%*CI*: 0.31–0.41), psychological symptoms (*ORs* = 3.17, 95%*CI*: 2.93–3.42), weekday video time (*ORs* = 1.10, 95%*CI*: 1.07–1.12), weekend video time (*ORs* = 1.13, 95%*CI*: 1.11–1.15), weekday video game time (*ORs* = 1.07, 95%*CI*: 1.04–1.09) and weekend video game time (*ORs* = 1.10, 95%*CI*: 1.08–1.12) were significantly associated with NSSI. Urban/rural residency and father’s education level were not significantly associated with NSSI.

### Associations sleep duration and NSSI and sex/ grade difference

After adjusting for covariates (variables that were significantly associated with each form of NSSI), multivariable logistic regressions was performed to explore the association between sleep duration and NSSI and sex/grade difference (see Tables 3 and 4). Compared to adolescents who reported 8–9 h of sleep, those who reported less than 6 h of sleep duration, including daily sleep duration (adjusted odds ratio [*aOR*] = 1.42, 95%*CI*: 1.18–1.71), weekday sleep (*aOR* = 1.24, 95%*CI*: 1.05–1.45), and weekend sleep (*aOR* = 1.55, 95%*CI*: 1.20–2.01), might have led to higher adjusted odds of NSSI for adolescents. Compared to adolescents who reported weekend catch-up sleep of 0–1 h, those who slept < 0 h (*aOR* = 1.38, 95%CI: 1.16–1.64) had a higher risk of NSSI. Sex and grade differences were found in the effects of sleep duration on NSSI. Males who reported ≥3 h of weekend catch-up sleep had significantly increased odds of NSSI (*aOR* = 1.20, 95%*CI*: 1.01–1.42). Senior middle school students who reported 1–2 h (*aOR* = 1.24, 95%*CI*: 1.01–1.52) or 2–3 h (*aOR* = 1.24, 95%*CI*: 1.02–1.51) of weekend catch-up sleep had significantly increased odds of NSSI. However, this result was not found in females and junior middle school students.

**Table 3:** Logistic regression of the effect of sleep duration on NSSI

Variables	NSSI	
	Crude *OR* (95%*CI*)	Adjusted *OR* (95%*CI*)
Daily sleep duration(h)		
8–9(ref)	1.00	1.00
< 6	1.78(1.51,2.11) ^**^	1.42(1.18,1.71) ^**^
6–7	1.21(1.09,1.34) ^**^	1.14(1.02,1.29) ^*^
7–8	1.11(1.01,1.22) ^*^	1.09(0.98,1.20)
9–10	0.99(0.85,1.16)	1.00(0.85,1.17)
≥ 10	0.93(0.72,1.20)	0.93(0.71,1.21)
Weekday sleep duration(h)		
8–9(ref)	1.00	1.00
< 6	1.58(1.37,1.83) ^**^	1.24(1.05,1.45) ^*^
6–7	1.15(1.04,1.28) ^*^	1.09(0.97,1.23)
7–8	1.16(1.05,1.28) ^*^	1.18(1.06,1.31) ^*^
9–10	1.05(0.90,1.23)	1.06(0.90,1.25)
≥ 10	0.94(0.75,1.19)	0.94(0.74,1.20)
Weekend sleep duration(h)		
8–9(ref)	1.00	1.00
< 6	2.10(1.65,1.68) ^**^	1.55(1.20,2.01) ^*^
6–7	1.31(1.11,1.56) ^*^	1.15(0.96,1.38)
7–8	1.16(1.01,1.32) ^*^	1.13(0.98,1.30)
9–10	1.01(0.91,1.12)	1.05(0.93,1.17)
≥ 10	1.03(0.94,1.13)	0.96(0.87,1.06)
Weekend catch-up sleep(h)		
0–1(ref)	1.00	1.00
< 0	1.69(1.43,1.99) ^**^	1.38(1.16,1.64) ^**^
1–2	1.01(0.89,1.14)	1.02(0.90,1.17)
2–3	1.14(1.01,1.29) ^*^	1.11(0.98,1.26)
≥ 3	1.25(1.11,1.41) ^**^	1.08(0.95,1.22)

* *P* <  0.05, ** *P* <  0.001; OR is odds ratios; CI is confidence interval; NSSI is non-suicidal self-injury; Adjusted model controlled regional areas, sex, grade, only child, parents’ education level, family economic status, sleep quality, psychological symptoms, weekday video time, weekend video time, weekday video game time, weekend video game time

**Table 4:** Adjusted OR (95% CI) of NSSI by sex and grade regrading sleep duration

Variables	NSSI [Adjusted *OR* (95%*CI*)]		NSSI [Adjusted *OR* (95%*CI*)]	
	Female	Male	Junior middle school	Senior middle school
Daily sleep duration(h)				
8–9(ref)	1.00	1.00	1.00	1.00
< 6	1.48(1.13,1.93) ^*^	1.38(1.07,1.78) ^*^	1.55(1.15,2.09) ^*^	1.30(1.01,1.67) ^*^
6–7	1.19(1.01,1.41) ^*^	1.10(0.94,1.30)	1.04(0.87,1.23)	1.13(0.94,1.35)
7–8	1.09(0.94,1.27)	1.08(0.94,1.25)	1.12(0.98,1.27)	1.00(0.84,1.19)
9–10	1.02(0.81,1.29)	0.99(0.80,1.22)	1.06(0.89,1.26)	0.80(0.51,1.26)
≥ 10	1.10(0.72,1.70)	0.85(0.61,1.19)	1.00(0.75,1.36)	0.78(0.44,1.38)
Weekday sleep duration(h)				
8–9(ref)	1.00	1.00	1.00	1.00
< 6	1.34(1.07,1.69) ^*^	1.13(0.90,1.43)	1.20(0.92,1.55)	1.28(1.01,1.62) ^*^
6–7	1.18(0.99,1.39)	1.03(0.88,1.21)	0.99(0.85,1.16)	1.16(0.96,1.41)
7–8	1.25(1.06,1.46) ^*^	1.12(0.96,1.30)	1.15(1.00,1.32) ^*^	1.18(0.97,1.44)
9–10	1.07(0.83,1.38)	1.06(0.85,1.31)	1.09(0.91,1.29)	1.04(0.56,1.94)
≥ 10	1.10(0.73,1.64)	0.87(0.64,1.18)	1.02(0.78,1.35)	0.76(0.44,1.32)
Weekend sleep duration(h)				
8–9(ref)	1.00	1.00	1.00	1.00
< 6	1.54(1.01,2.35) ^*^	1.59(1.14,2.20) ^*^	1.20(0.78,1.86)	1.74(1.26,2.40) ^*^
6–7	1.29(0.97,1.72)	1.09(0.86,1.38)	1.39(1.06,1.82) ^*^	0.97(0.76,1.24)
7–8	1.12(0.91,1.37)	1.14(0.94,1.39)	1.24(1.01,1.53) ^*^	1.04(0.86,1.26)
9–10	0.98(0.84,1.14)	1.12(0.95,1.31)	1.14(0.98,1.33)	0.97(0.83,1.14)
≥ 10	0.94(0.82,1.08)	0.98(0.85,1.12)	1.04(0.91,1.19)	0.90(0.78,1.03)
Weekend catch-up sleep				
0–1(ref)	1.00	1.00	1.00	1.00
< 0	1.29(0.97,1.72)	1.49(1.20,1.85) ^**^	1.15(0.92,1.43)	1.92(1.45,2.54) ^**^
1–2	0.96(0.79,1.17)	1.08(0.90,1.30)	0.89(0.75,1.06)	1.24(1.01,1.52) ^*^
2–3	1.10(0.91,1.32)	1.11(0.94,1.32)	1.02(0.86,1.20)	1.24(1.02,1.51) ^*^
≥ 3	0.97(0.81,1.17)	1.20(1.01,1.42) ^*^	1.06(0.89,1.26)	1.15(0.95,1.38)

* *P* <  0.05, ** *P* <  0.001; OR is odds ratios; CI is confidence interval; NSSI is non-suicidal self-injury; Adjusted model controlled regional areas, sex, grade, only child, parents’ education level, family economic status, sleep quality, psychological symptoms, weekday video time, weekend video time, weekday video game time, weekend video game time

### Associations sleep duration and total NSSI number

**Fig. 1:**
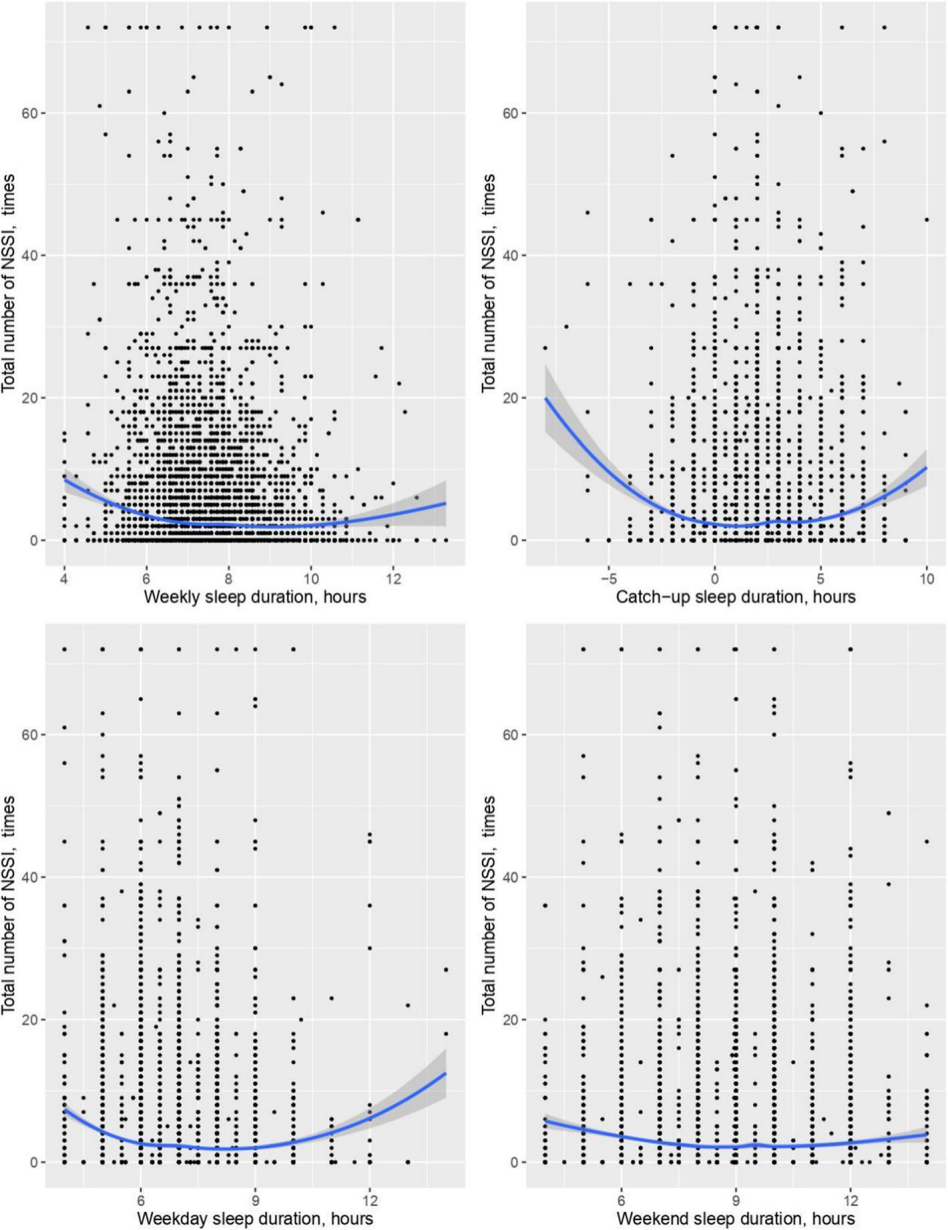
Association between sleep duration and total NSSI number

Scatterplots fit with a LOESS curve depicting the relationship between sleep duration and total NSSI number were non-linear (Fig. 1). Daily sleep, weekend catch-up sleep, weekday sleep and weekend sleep duration all had a U-shaped relationship with the total NSSI number.

A GetData Graph Digitizer was used to determine the nadir point. The U-shape curve depicting the relationship between daily sleep duration and NSSI had a nadir point of approximately 8.2 h, the weekend catch-up sleep U-shaped curve was seen with a nadir point of approximately 0.9 h, the weekday sleep U-shaped curve was seen with a nadir point of approximately 7.7 h, and the weekend sleep U-shaped curve was seen with a nadir point of approximately 8.2 h.

After adjusting for covariates (regional areas, sex, grade, registered residence, household structure, parents’ education level, self-perceived family economic status, sleep quality, psychological symptoms, and screen time), binomial regression was performed to test the association between sleep duration and total NSSI number (see Table 5). The results of the binomial regression analysis showed that a positive U-shaped association was observed between the entire sleep duration and total NSSI number (*P* <  0.001).

**Table 5:** Binomial regression analysis of the effect of sleep duration on total NSSI number

Variables	Total NSSI number					
	Crude *OR(95%CI)*	*F*	*P* value	Adjusted *OR*(95%CI)	*F*	*P* value
Total number of NSSI						
Daily sleep (h)	−4.01(−4.90, −3.12)^**^	63.68	< 0.001	−2.76(−3.62, −1.91) ^**^	88.36	< 0.001
Daily sleep (h) ^2^	0.23(0.18,0.29) ^**^			0.17(0.11,0.22) ^**^		
Total number of NSSI						
Weekday sleep (h)	−3.91(−4.58,-3.24) ^**^	86.48	< 0.001	−2.59(−3.25,-1.94) ^**^	89.48	< 0.001
Weekday sleep (h) ^2^	0.24(0.20,0.29) ^**^			0.17(0.12,0.21) ^**^		
Total number of NSSI						
Weekend sleep (h)	−2.14(−2.61,-1.67)^**^	42.74	< 0.001	−1.14(−1.60,-0.69) ^**^	86.95	< 0.001
Weekend sleep (h) ^2^	0.11(0.09,0.14) ^**^			0.06(0.03,0.08) ^**^		
Total number of NSSI						
catch-up sleep (h)	−0.36(−0.47,-0.26) ^**^	52.88	< 0.001	−0.24(−0.34,-0.15) ^**^	87.36	< 0.001
catch-up sleep (h) ^2^	0.10(0.08,0.12) ^**^			0.05(0.04,0.07) ^**^		

* *P* < 0.05, ** *P* < 0.001; OR is odds ratios; CI is confidence interval; NSSI is non-suicidal self-injury; Adjusted model controlled regional areas, sex, grade, registered residence, only child, parents’ education level, family economic status, sleep quality, psychological symptoms, weekday video time, weekend video time, weekday video game time, weekend video game time

## Discussion

The present study examined the relationship between sleep duration and NSSI among adolescents in China. As hypothesized, our research found that both sleep deprivation and oversleeping were associated with an increased risk of NSSI after adjustment for important confounding factors. We believe that this is the first large-sample cross-sectional study to report a U-shaped association between sleep duration and total NSSI number.

In this study, we found that sleep deprivation and oversleeping have become increasingly prevalent in China, with 68.5% of adolescents sleeping 8 h or fewer on school days and 37.8% of adolescents sleeping 10 h or more on weekends. Meanwhile, large discrepancies between weekday versus weekend sleep duration was also common among adolescents, and in our study the average weekend catch-up sleep duration of adolescents was 1.9 h. Similar results were obtained in previous studies [14, 22]. While adolescent changes in the sleep-wake pattern might be explained by their unique physiological characteristics, evidence suggests that sleep patterns in adolescents are influenced by circadian and homeostatic systems, at least in part [39]. The circadian system, a hierarchically organized network of structures responsible for generating approximately 24-h rhythms, is driven in mammals by a circadian pacemaker located in the suprachiasmatic nuclei (SCN) of the hypothalamus [40]. The homeostatic process regulates sleep pressure which accumulates with wake duration and dissipates during subsequent sleep [41]. Previous studies suggest that the sensitivity of the circadian timing system to light exposure may differ in adolescence, favoring a blunted phase advance response to light exposure in the morning and an exaggerated phase-delay response to light exposure in the evening [42]. Evidence also indicates that the internal day length may be longer in adolescents than in adults, thus contributing to phase delay [16]. In addition, melatonin is an important mediator in the circadian system. Adolescents experience a lower amplitude of the daily rhythm of melatonin secretion, which may in turn dampen the signal for sleep [16]. Furthermore, the homeostatic and circadian regulation of sleep is sensitive to gonadal hormones, and these hormones are necessary for the development of a delayed phase during adolescence [39]. These physiological changes may be part of the reason why adolescents sleep too much or too little. However, adolescent sleep is also affected by the psychosocial environment. In a survey of 1101 students aged 13–16 years in Australia, Vernon et al. found that increased nighttime mobile phone use was directly associated with increased occurrences of poor sleep behavior [43]. In another study, Buzek et al. reported a negative association between sleep duration and academic stress among children from families with low socioeconomic status [44]. Most students reported waking up before their natural biological rise time due to early school starting times. In a study of Singaporean female students, a greater increase in sleep duration on school nights and lower levels of subjective sleepiness were reported at 1 month after implementing a 45-min delay in school start times. These positive changes were maintained at 9 months [45]. In the present study, we found that insufficient sleep duration occurred more often among females than males, with approximately 63.5% of males and 73.1% of females reported sleeping for less than 8 h on weeknights. The results obtained in this study are consistent with most other findings [46–48], although some studies have found no sex-related differences in sleep duration [49, 50]. Differences across sex in this study could be due to females requiring more preparation time in the morning, and advanced onset of puberty in females may contribute to later bedtimes [48, 51, 52]. The results of the present study also suggests that 49.6% of junior middle school students and 87.6% of senior middle school students reported sleeping less than 8 h on school days. From these results, the difference observed among different grade groups can be partly explained by the following facts: first, bioregulatory pressures sustain evening alertness later into the night with increasing age; second, parental supervision and time spent on sports/physical training largely reduce, academic demands increase, and social networks expand with increasing age, so there is an increased risk for insufficient sleep among senior middle school students [53]. The reasons for variable sleep duration across sex or grade require careful consideration in future studies, as they may be targets of educational or other interventions.

In recent years, the prevalence rates of NSSI were high and results from the present study showed that the 12-month prevalence rate of NSSI was 29.4%. Until now, however, few studies have examined the association between sleep duration and NSSI. Previous studies have shown a close link between sleep problems and self-harm; difficulties initiating sleep, early morning wakening, short sleep duration, severe sleep complaints, daytime sleepiness, and nightmares were associated with an increased risk of self-harm in a dose-dependent manner [28, 29, 54, 55]. In this study, after controlling for potential confounding factors, we found that compared to males who reported weekend catch-up sleep of 0–1 h, males who slept < 0 h or ≥ 3 h had a higher risk of NSSI. The results of this study indicate that either excessive or restricted weekend catch-up sleep among males was associated with an increased risk of NSSI. Furthermore, we found that daily sleep, weekend catch-up sleep, weekday sleep, and weekend sleep duration had a U-shape relationship with total NSSI number. However, the underlying mechanism remains unclear. There are several possible explanations for this. It seems plausible that improper sleep duration may disrupt the circadian clock, leading to a subsequent increase in inflammatory biomarkers. In addition, inflammatory markers have been shown to be key markers associated with NSSI. Many studies suggest that the extreme of long sleep duration/shorter sleep duration or circadian misalignment significantly increased plasma tumor necrosis factor-alpha (TNF-α), interleukin 10 (IL-10) and C-reactive protein (CRP) [56, 57]. Increased inflammation might change major neurotransmitter metabolism, which subsequently affects frontal function and decreases response inhibition. Additionally, NSSI was related to greater behavioral impulsivity and increased serum TNF-α levels. Therefore, frontal dysfunction associated with greater inflammation might explain the neurobiological basis of NSSI [58]. The hypothalamic-pituitary-adrenal (HPA) axis seems to be a potential mechanism that may explain the link between sleep deprivation and NSSI. Previous findings suggest that total sleep deprivation and chronic circadian misalignment differentially influence cortisol levels. Acute total sleep deprivation increases cortisol levels, whereas circadian misalignment decreases cortisol levels. The current research results are not consistent with the effect of the HPA axis on NSSI. Reichl et al. reported that adolescents engaging in NSSI exhibited significantly higher cortisol awakening responses compared to healthy controls [59]. However, another study showed that the HPA axis is hyporesponsive in adolescents with NSSI. Therefore, reduced secretion of cortisol could play a role in promoting the vulnerability of these individuals to acute stress and maladaptive stress responses [60]. Furthermore, sleep deprivation and NSSI may be linked to reduced connectivity in the default mode network (DMN). Irregular sleep patterns among adolescents are associated with increased path length within the DMN specifically in the right and left lateral parietal lobule, suggesting that sleep regularity may be vital for optimal brain functioning during this developmental period [61]. In addition, reduced DMN connectivity is often observed among adolescents with neuropsychiatric conditions, such as attention-deficit hyperactivity disorder (ADHD), depression, and emerging psychosis [62]. Therefore, sleep irregularity may affect NSSI through alterations in brain connectivity.

This study expands our knowledge of the association between sleep duration and NSSI by examining sex and grade differences. We believe the present study is the first to show a U-shaped association between sleep duration and total NSSI number. Another major strength of this investigation was the large representative sample and age range studied, which included late childhood through adolescence. In addition, we considered a wide range of potential covariates, including sleep quality, psychological symptoms, and screen time. We believe this study provides key insights for the prevention of adolescent NSSI.

The present study had several limitations. First, this study was cross-sectional, thus causality cannot be inferred. However, most of the previous studies on NSSI used cross-sectional research [63, 64], and our findings pertaining to the association between sleep and NSSI were similar to those in previous cohort studies [54, 65, 66]. Further longitudinal studies are needed to replicate our findings and clarify the causality and mechanisms that relate to sleep duration, neuropsychiatric status, and NSSI. Second, self-reported NSSI is prone to recall bias and social desirability bias. For a majority of adolescents, self-harm remains a hidden act [67]. Investigations into NSSI are sensitive, and the reporting rate may be lower than the actual levels [68]. In addition, sleep duration data were obtained by self-report and were not verified by objective measures such as actigraphy (a measurement of the motor activity sensor) or polysomnography (sleep study) because it was a nationwide large-scale survey study. The collection of objective sleep measures via physiologic monitoring is cost-prohibitive and often not feasible. Additionally, self-reported sleep duration may be an overestimation of the actual measured sleep duration [69, 70]. However, previous studies have shown good agreement between self-reported sleep durations and those obtained through actigraphic monitoring [69, 70]. Moreover, self-reported sleep duration is considered more accurate in detecting long-term sleep habits and more suitable for large-scale epidemiologic studies [71]. In the future, measurement of sleep duration using polysomnography or actigraphy may be recommended to explore the mechanism further. Third, while we investigated students’ screen time in our study, we ignored students’ social media use. This needs to be investigated more thoroughly in the near future. Fourth, the association between sleep duration and NSSI may be partly accounted for by other parameters such as childhood physical or sexual abuse. Future studies are needed to examine mediators and moderators between sleep duration and NSSI in adolescents, which would provide more clues on adolescent NSSI prevention. Lastly, studies with a larger sample size may have increased the generalizability of the results and powered to detect even small differences. However, statistically significant did not necessarily mean clinically significant, thus the clinical or practical implications of our findings should be studied further.

## Conclusion

This study provides key insights into the association between sleep duration and NSSI among Chinese adolescents. Our findings show that excessive or restricted sleep duration is associated with an increased risk of NSSI. Parents, teachers, and child health workers are encouraged to be vigilant and screen for sleep problems in adolescents. Future research will determine if early intervention with sleep duration reduces the risk of NSSI in adolescents.

## Supplementary Information

**Additional file 1.** English version and Chinese version of Chinese Adolescent Physical and Mental Health Monitoring Questionnaire.

## Data Availability

The datasets that were generated analyzed for the current study are not publicly available as the author does not have permission to share the data.

## Abbreviations

NSSI: Non-Suicidal Self-Injury
ORs: Crude Odds Ratios
aOR: Adjusted Odd Ratio
CI: Confidence Interval
SCN: Suprachiasmatic Nuclei
TNF-α: Tumor Necrosis Factor-alpha
IL-10: Interleukin 10
CRP: C-Reactive Protein
HPA: Hypothalamic-Pituitary-Adrenal
DMN: Default Mode Network
LOESS: Locally Estimated Scatterplot Smoothing
ADHD: Attention-Deficit Hyperactivity Disorder
MSQA: Multidimensional Sub-health Questionnaire of Adolescents
