# Supplementary material for: Nonlinear relationship between sleep duration and non-suicidal self-injurious behaviour among Chinese adolescents
Source: BMC Psychiatry. 2021 Oct 21;21:521. doi: 10.1186/s12888-021-03539-x (PMC8532314; doi:10.1186/s12888-021-03539-x)
Supplement: Supplementary file 1 — Additional file 1. English version and Chinese version of Chinese Adolescent Physical and Mental Health Monitoring Questionnaire. [file 12888_2021_3539_MOESM1_ESM.docx]

**Chinese Adolescent Physical and Mental Health Monitoring Questionnaire**

The purpose of this survey is to understand the physical and mental health status and lifestyle of teenagers. Please tick or fill in the corresponding answer according to your actual situation and real experience. Your objective and true answer will be very helpful for us to develop measures to promote the healthy growth of teenagers. We sincerely thank you for your cooperation! This survey uses an anonymous method, and all information is strictly confidential, please do not have any concerns.

**Socio-demographic Information**

1. Gender ① male; ② female
2. School __________________
3. Grade ① First grade; ② Second grade; ③ Third grade;
4. Date of birth__________________
5. Residence ① rural area; ② urban area
6. Are you an only child? ① Yes; ② No
7. Education level of your father (including adoptive father and stepfather)
8. No father;
9. Father has not graduated from primary school or attended school;
10. Graduated from primary school;
11. Junior middle school;
12. Senior middle school or technical secondary school;
13. College or more
14. Education level of your mother (including foster mother and stepmother)
    1. No mother;

② Mother has not graduated from primary school or attended school;

③ Graduated from primary school;

④ Junior middle school;

⑤ Senior middle school or technical secondary school;

⑥ College or more

1. Compared with other students, how do you evaluate your family's economic conditions?
   1. Poor; ② Very poor; ③ Moderate; ④ Very good; ⑤ Good

**Psychological Symptoms**

The following table lists psychological, social, energy and other discomforts, please read each one carefully, and then choose the one that best suits you based on your actual feelings in the last 3 months. Tick in the options (√).

| Questions | lasting  ≥3 months | lasting ≥2 months | lasting  ≥1 month | lasting  ≥2 weeks | lasting ≥1 week | lasting ＜1 week |
| --- | --- | --- | --- | --- | --- | --- |
| 1. Not enjoy anything at all | 1 | 2 | 3 | 4 | 5 | 6 |
| 2. Often feel nervous | 1 | 2 | 3 | 4 | 5 | 6 |
| 3. Often blame yourself | 1 | 2 | 3 | 4 | 5 | 6 |
| 4. Unable to relax when feeling tense | 1 | 2 | 3 | 4 | 5 | 6 |
| 5. Indecision about doing things | 1 | 2 | 3 | 4 | 5 | 6 |
| 6. Often feel upset | 1 | 2 | 3 | 4 | 5 | 6 |
| 7. Often afraid of empty places | 1 | 2 | 3 | 4 | 5 | 6 |
| 8. Unprovoked fear | 1 | 2 | 3 | 4 | 5 | 6 |
| 9. Always feel hopeless | 1 | 2 | 3 | 4 | 5 | 6 |
| 10. Unable to concentrate | 1 | 2 | 3 | 4 | 5 | 6 |
| 11. Often feel that someone is talking about me | 1 | 2 | 3 | 4 | 5 | 6 |
| 12. I always feel that others can know my private thoughts | 1 | 2 | 3 | 4 | 5 | 6 |
| 13. Often feel depressed | 1 | 2 | 3 | 4 | 5 | 6 |
| 14. There are always unnecessary thoughts or words in the mind | 1 | 2 | 3 | 4 | 5 | 6 |
| 15. Think of death repeatedly | 1 | 2 | 3 | 4 | 5 | 6 |
| 16. Often think about how to commit suicide (such as how to take poison, cut the wrist, jump off the building, etc.) | 1 | 2 | 3 | 4 | 5 | 6 |
| 17. Often afraid of going to public places | 1 | 2 | 3 | 4 | 5 | 6 |
| 18. Always have the urge to throw things | 1 | 2 | 3 | 4 | 5 | 6 |
| 19. Feel uncomfortable in crowded places | 1 | 2 | 3 | 4 | 5 | 6 |
| 20. Feel like everyone’s against you | 1 | 2 | 3 | 4 | 5 | 6 |
| 21. Always feel nervous when alone | 1 | 2 | 3 | 4 | 5 | 6 |
| 22. Always feel that most people cannot be trusted | 1 | 2 | 3 | 4 | 5 | 6 |
| 23. Often argue with others | 1 | 2 | 3 | 4 | 5 | 6 |
| 24. I always don't like talking about problems with classmates and friends | 1 | 2 | 3 | 4 | 5 | 6 |
| 25. Often loses temper uncontrollably | 1 | 2 | 3 | 4 | 5 | 6 |
| 26. Always hate going to school | 1 | 2 | 3 | 4 | 5 | 6 |
| 27. Bad mental state all day | 1 | 2 | 3 | 4 | 5 | 6 |
| 28. I always feel restless when I hear about the exam | 1 | 2 | 3 | 4 | 5 | 6 |
| 29. Always worry about the teacher asking yourself questions in class | 1 | 2 | 3 | 4 | 5 | 6 |
| 30. Often angry about little things | 1 | 2 | 3 | 4 | 5 | 6 |
| 31. It is almost difficult to study at home | 1 | 2 | 3 | 4 | 5 | 6 |
| 32. Compared with my classmates, I often find it difficult to study | 1 | 2 | 3 | 4 | 5 | 6 |
| 33. I often feel uneasy when others look at me | 1 | 2 | 3 | 4 | 5 | 6 |
| 34. It's always difficult to remember what you learned recently | 1 | 2 | 3 | 4 | 5 | 6 |
| 35. Feel uncomfortable in school life | 1 | 2 | 3 | 4 | 5 | 6 |
| 36. It is always difficult to adapt to the teacher's teaching method | 1 | 2 | 3 | 4 | 5 | 6 |
| 37. When I feel down, I often don’t want to talk to others | 1 | 2 | 3 | 4 | 5 | 6 |
| 38. I often feel that people are unfriendly to me and don't like me | 1 | 2 | 3 | 4 | 5 | 6 |
| 39. Won’t seek for help when in trouble | 1 | 2 | 3 | 4 | 5 | 6 |

**Screen Time**

1. On an average school day, how many hours do you watch video (such as watching TV, mobile phone, MP4, DVD/VCD) ?

① I do not watch video on an average school day

- 1. ≤0.5 h
  2. 0.5–1 h
  3. 1–2 h
  4. 2–4 h
  5. 4–6 h
  6. ＜6 h

1. On an average weekend, how many hours do you watch video (such as watching TV, mobile phone, MP4, DVD/VCD) ?

① I do not watch video on weekend

② ≤0.5 h

③ 0.5–1 h

④ 1–2 h

⑤ 2–4 h

⑥ 4–6 h

⑦ ＜6 h

3. On an average school day, how many hours do you play video games (such as game consoles, computer games, mobile games)?

① I do not play video games on an average school day

② ≤0.5 h

③ 0.5–1 h

④ 1–2 h

⑤ 2–4 h

⑥ 4–6 h

⑦ ＜6 h

4. On an average weekend, how many hours do you play video games (such as game consoles, computer games, mobile games)?

① I do not play video games on weekend

② ≤0.5 h

③ 0.5–1 h

④ 1–2 h

⑤ 2–4 h

⑥ 4–6 h

⑦ ＜6 h

**Sleep Variables**

1. In the last month, how many hours of actual sleep do you usually get at night on weekdays (Not including time spent lying in bed and unable to sleep)? ­­­­­­­­__________________

2. In the last month, how many hours of actual sleep do you usually get at night on weekend (Not including time spent lying in bed and unable to sleep)? ­­­­­­­­__________________

3. In the past month, what do you think of your sleep quality?

1. Good; ② Very good; ③ Poor; ④ Very poor

**NSSI**

**In the past 12 months, have you ever harmed yourself in a way that was deliberate, but not intended to take your life?**

| **Behavior** | **YES/ NO** | |
| --- | --- | --- |
| 1. Have you ever hit yourself? | YES,  There are ­times; | NO |
| 2. Have you ever pulled your hair yourself? | YES,  There are ­times; | NO |
| 3. Have you ever banged your head or fist against something? | YES,  There are ­times; | NO |
| 4. Have you ever pinched or scratched yourself? | YES,  There are ­times; | NO |
| 5. Have you ever bitten yourself? | YES,  There are ­times; | NO |
| 6. Have you ever cut or pierced yourself | YES,  There are ­times; | NO |
| 7. Have you ever deliberately taken an overdose (e.g. of drugs, alcohol or smoking) | YES,  There are ­times; | NO |
| 8. Have you ever ingested a toxic substance or object? | YES,  There are ­times; | NO |

**中国青少年身心健康监测问卷**

本次调查是为了解青少年的身心健康状况和生活行为方式，请根据你的实际情况与真实体验在相应的答案上划一个“√”号或者填上相应的回答。你客观、真实的回答，将对我们制定促进青少年健康成长的措施有很好的帮助，真诚感谢你的合作！本调查采用无记名方式，并且所有信息严格保密，请不要有任何顾虑。

一般情况

**1. 性别** ① 男； ② 女

**2. 学校**

**3. 年级** ① 高一； ② 高二； ③ 高三；

**4. 出生日期** 年 月 日

**5. 户口所在地**

① 农村；

② 城镇

**6. 你是否为独生子女**

① 是；

② 否

**7. 你父亲（包括养父、继父）的文化程度**

① 没有父亲；

② 父亲小学未毕业或没有上过学；

③ 小学毕业；

④ 初中；

⑤ 高中或中专；

⑥ 大专或大专以上

**8．你母亲（包括养母、继母）的文化程度**

① 没有母亲；

② 母亲小学未毕业或没有上过学；

③ 小学毕业；

④ 初中；

⑤ 高中或中专；

⑥ 大专或大专以上

**9．你认为你的家庭经济条件与其他同学相比属于**

① 差；

② 较差；

③ 中等；

④ 较好；

⑤ 好

**情绪状况**

以下表格中列出了有些人可能会有心理、社会、活力等不适情况，请仔细阅读每一条,然后根据**最近 3 个月以来**自己的实际感受，选择最符合您的一种情况，在后面相应的选项中划（**√**）。

| **题 目** | | **持续3个月以上** | **持续**  **2个月以上** | **持续**  **1个月以上** | **持续**  **2星期以上** | **持续**  **1星期以上** | **没有或持续不到1星期** |
| --- | --- | --- | --- | --- | --- | --- | --- |
|  | 对事物不感兴趣 | **1** | **2** | **3** | **4** | **5** | **6** |
|  | 常常感到紧张 | **1** | **2** | **3** | **4** | **5** | **6** |
|  | 经常责怪自己 | **1** | **2** | **3** | **4** | **5** | **6** |
|  | 常常感到坐立不安、心神不定 | **1** | **2** | **3** | **4** | **5** | **6** |
|  | 做事经常犹豫不决 | **1** | **2** | **3** | **4** | **5** | **6** |
|  | 常常感到心里烦躁 | **1** | **2** | **3** | **4** | **5** | **6** |
|  | 常常害怕空旷的场所 | **1** | **2** | **3** | **4** | **5** | **6** |
|  | 经常会无缘无故地感到害怕 | **1** | **2** | **3** | **4** | **5** | **6** |
|  | 总是感到前途没有希望 | **1** | **2** | **3** | **4** | **5** | **6** |
|  | 注意力无法集中 | **1** | **2** | **3** | **4** | **5** | **6** |
|  | 常常感到有人在谈论我 | **1** | **2** | **3** | **4** | **5** | **6** |
|  | 总是感觉旁人能知道我的私下想法 | **1** | **2** | **3** | **4** | **5** | **6** |
|  | 常常感到苦闷 | **1** | **2** | **3** | **4** | **5** | **6** |
|  | 头脑中总是有不必要的想法或字句盘  旋 | **1** | **2** | **3** | **4** | **5** | **6** |
|  | 反复想到死 | **1** | **2** | **3** | **4** | **5** | **6** |
|  | 经常想到怎样去实施自杀（如怎样去  服毒、割腕、跳楼等） | **1** | **2** | **3** | **4** | **5** | **6** |
|  | 常常害怕去公共场合 | **1** | **2** | **3** | **4** | **5** | **6** |
|  | 经常有想摔东西的冲动 | **1** | **2** | **3** | **4** | **5** | **6** |
|  | 在人多的地方感到不自在 | **1** | **2** | **3** | **4** | **5** | **6** |
|  | 总觉得别人在跟我作对 | **1** | **2** | **3** | **4** | **5** | **6** |
|  | 单独一个人时总是感觉精神很紧张 | **1** | **2** | **3** | **4** | **5** | **6** |
|  | 总是感到大多数人都不可信任 | **1** | **2** | **3** | **4** | **5** | **6** |
|  | 经常与人争论，抬杠 | **1** | **2** | **3** | **4** | **5** | **6** |
|  | 总是不喜欢和同学、朋友在一起谈论  问题 | **1** | **2** | **3** | **4** | **5** | **6** |
|  | 经常不能控制地大发脾气 | **1** | **2** | **3** | **4** | **5** | **6** |
|  | 总是很讨厌上学 | **1** | **2** | **3** | **4** | **5** | **6** |
|  | 一天到晚对什么都提不起精神 | **1** | **2** | **3** | **4** | **5** | **6** |
|  | 一听说要考试，总感到坐立不安 | **1** | **2** | **3** | **4** | **5** | **6** |
|  | 上课时总是担心老师提问自己 | **1** | **2** | **3** | **4** | **5** | **6** |
|  | 经常因一些小事而愤怒 | **1** | **2** | **3** | **4** | **5** | **6** |
|  | 在家里几乎很难安心学习 | **1** | **2** | **3** | **4** | **5** | **6** |
|  | 与同学相比，我常常感到学习很困难 | **1** | **2** | **3** | **4** | **5** | **6** |
|  | 当别人看着我时，常常感到十分紧张 | **1** | **2** | **3** | **4** | **5** | **6** |
|  | 近一段时间，总是很难记住学习内容 | **1** | **2** | **3** | **4** | **5** | **6** |
|  | 对现在的学校生活常常感到不适应 | **1** | **2** | **3** | **4** | **5** | **6** |
|  | 总是很难适应老师的教学方法 | **1** | **2** | **3** | **4** | **5** | **6** |
|  | 当我心情低落时，常常不愿向其他人  倾诉 | **1** | **2** | **3** | **4** | **5** | **6** |
|  | 常常感到人们对我不友好，不喜欢我 | **1** | **2** | **3** | **4** | **5** | **6** |
|  | 当遇到困难时，大多不想去求助于别人 | **1** | **2** | **3** | **4** | **5** | **6** |

**视屏时间**

**1. 上学日，你每天坐着或躺着看视频的时间？（如看电视、手机、MP4、DVD/VCD）**

① 没有；

② ≤30 分钟； ③ 30 分钟～1 小时； ④ 1～2 小时；

⑤ 2～4 小时； ⑥ 4～6 小时；

⑦ ＞6 小时

**2. 周末，你每天坐着或躺着看视频的时间？（如看电视、手机、MP4、DVD/VCD）**

① 没有；

② ≤30 分钟； ③ 30 分钟～1 小时； ④ 1～2 小时；

⑤ 2～4 小时； ⑥ 4～6 小时；

⑦ ＞6 小时

**3. 上学日，你每天用于玩游戏或玩电脑的时间？（如游戏机、电脑游戏、手机游戏）**

① 没有；

② ≤30 分钟； ③ 30 分钟～1 小时； ④ 1～2 小时；

⑤ 2～4 小时； ⑥ 4～6 小时；

⑦ ＞6 小时

**4. 周末，你每天用于玩游戏或玩电脑的时间？（如游戏机、电脑游戏、手机游戏）**

① 没有；

② ≤30 分钟； ③ 30 分钟～1 小时； ④ 1～2 小时；

⑤ 2～4 小时； ⑥ 4～6 小时；

⑦ ＞6 小时

**睡眠情况**

**1. 近 1 个月，在上学日，你每天一般实际睡 小时（不包括躺在床上睡不着的时间）**

**2. 近 1 个月，在周末，你每天一般实际睡 小时（不包括躺在床上睡不着的时间）**

**3. 近 1 个月，你认为自己总的睡眠质量** ①很好 ②较好 ③较差 ④很差

**NSSI**

**最近1年内，你有没有以下故意伤害自己的行为？（不以自杀为目的）**

| **行 为** | **发生情况** |
| --- | --- |
| 1. 故意打自己 | ①有 次；　　②没有 |
| 2. 故意拽头发 | ①有 次；　　②没有 |
| 3. 故意撞头或用拳头击打其他物体 | ①有 次；　　②没有 |
| 4. 故意掐自己或抓伤自己 | ①有 次；　　②没有 |
| 5. 故意咬伤自己 | ①有 次；　　②没有 |
| 6. 故意割伤或刺伤自己 | ①有 次；　　②没有 |
| 7. 故意过量服用药物、饮酒或吸烟等 | ①有 次；　　②没有 |
| 8. 故意吞食异物（无法消化的物品） | ①有 次；　　②没有 |
